# Novel *PHOTOPERIOD-1* gene variants associate with yield-related and root-angle traits in European bread wheat

**DOI:** 10.1007/s00122-024-04634-9

**Published:** 2024-05-10

**Authors:** Manar Makhoul, Rica-Hanna Schlichtermann, Samson Ugwuanyi, Sven E. Weber, Kai P. Voss-Fels, Andreas Stahl, Holger Zetzsche, Benjamin Wittkop, Rod J. Snowdon, Christian Obermeier

**Affiliations:** 1 Department of Plant Breeding, Justus Liebig University Giessen, Giessen, Germany; 2 Institute for Grapevine Breeding, Hochschule Geisenheim University, Geisenheim, Germany; 3 Institute for Resistance Research and Stress Tolerance, Julius Kühn Institute, Quedlinburg, Germany

## Abstract

**Key message:**

*PHOTOPERIOD-1* homoeologous gene copies play a pivotal role in regulation of flowering time in wheat. Here, we show that their influence also extends to spike and shoot architecture and even impacts root development.

**Abstract:**

The sequence diversity of three homoeologous copies of the *PHOTOPERIOD-1* gene in European winter wheat was analyzed by Oxford Nanopore amplicon-based multiplex sequencing and molecular markers in a panel of 194 cultivars representing breeding progress over the past 5 decades. A strong, consistent association with an average 8% increase in grain yield was observed for the PpdA1-Hap1 haplotype across multiple environments. This haplotype was found to be linked in 51% of cultivars to the 2NS/2AS translocation, originally introduced from *Aegilops ventricosa*, which leads to an overestimation of its effect. However, even in cultivars without the 2NS/2AS translocation, PpdA1-Hap1 was significantly associated with increased grain yield, kernel per spike and kernel per m^2^ under optimal growth conditions, conferring a 4% yield advantage compared to haplotype PpdA1-Hap4. In contrast to *Ppd-B1* and *Ppd-D1*, the *Ppd-A1* gene exhibits novel structural variations and a high number of SNPs, highlighting the evolutionary changes that have occurred in this region over the course of wheat breeding history. Additionally, cultivars carrying the photoperiod-insensitive *Ppd-D1a* allele not only exhibit earlier heading, but also deeper roots compared to those with photoperiod-sensitive alleles under German conditions. PCR and KASP assays have been developed that can be effectively employed in marker-assisted breeding programs to introduce these favorable haplotypes.

**Supplementary Information:**

The online version contains supplementary material available at 10.1007/s00122-024-04634-9.

## Introduction

In wheat, the photoperiod genes (*Ppd-1*) have been found to play a critical role in the regulation of flowering time and adaptability (Snape et al. 2001; Yang et al. 2009; Kitagawa et al. 2012; Kamran et al. 2014; Alvarez et al. 2023). The photoperiod response is primarily controlled by three major genes: *Ppd-A1*, *Ppd-B1*, and *Ppd-D1*, located on homoeologous chromosomes on the short arm of chromosomes 2 (Law et al. 1978; Scarth and Law 1983, 1984; McIntosh et al. 2003). Photoperiod-sensitive (PS) plants carry an ancestral allele of the *Ppd-1* genes and exhibit a significant delay in flowering under short-day conditions. Conversely, photoperiod-insensitive (PI) plants are characterized by mutations in the *Ppd-1* genes, resulting in accelerated heading regardless of whether they are exposed to short or long-day conditions.

Previous investigations have identified distinct genetic variations associated with photoperiod insensitivity. The photoperiod-insensitive alleles *Ppd-D1a* and *Ppd-A1a* exhibit large deletions within their promoter regions (Beales et al. 2007; Wilhelm et al. 2009; Nishida et al. 2013), while the photoperiod-insensitive *Ppd-B1a* allele has been linked to an increased copy number, leading to elevated gene expression levels and conferring a photoperiod-insensitive phenotype (Díaz et al. 2012; Cane et al. 2013; Würschum et al. 2015, 2019). Additionally, Sun et al. (2014) demonstrated that the transcriptional levels of the *Ppd-B1* gene are modulated by DNA methylation, revealing its correlation with photoperiod insensitivity. These photoperiod-insensitive alleles exhibit variable effects on flowering acceleration, with *Ppd-D1a* generally exhibiting the strongest effect, followed by *Ppd-B1a* and *Ppd-A1a* (Wilhelm et al. 2009; Nishida et al. 2013; Díaz et al. 2012; Shaw et al. 2012; Bentley et al. 2011, 2013). Furthermore, Beales et al. (2007) identified three loss-of-function alleles of the *Ppd-1* genes. These alleles were characterized by a large insertion of 4819 bp and 5 bp deletion in the first intron and exon 7 of *Ppd-D1,* respectively*,* as well as a deletion of 303 bp spanning from the middle of exon 5 to the middle of exon 6 of *Ppd-A1*. Guo et al. (2010) and Shaw et al. (2013) demonstrated that loss-of-function *Ppd-D1* alleles were distributed widely across the globe; however, the phenotypic effects resulting from loss-of-function alleles remain unclear and require further experimental testing.

Numerous studies investigated the impact of *Ppd-1* genes on various aspects of plant development. It has been found that *Ppd-1* genes strongly influence on yield-related traits in wheat, including the number of kernel-producing spikelets, spike length, plant height, leaf size, thousand grain weight and spike fertility (Tanio and Kato 2007; Seki et al. 2011, 2013; Langer et al. 2014; Kiss et al. 2014; Cho et al. 2015; Zhang et al. 2015; Boden et al. 2015; Grogan et al. 2016; Steinfort et al. 2017b; Jones et al. 2017; Prieto et al. 2018; Ramirez et al. 2018; Arjona et al. 2018, 2020; Ochagavía et al. 2018; Pérez-Gianmarco et al. 2019, 2020; Sun et al. 2020; Amo et al. 2022; Fait and Balashova 2022; Errum et al. 2023). However, the majority of these studies focused on the impact of insensitive and sensitive alleles on agronomic traits, identifying structural variations within the *Ppd-1* region in bread wheat populations using PCR-based methods to detect fragment size differences. Due to the strong effects of major mutations in key flowering-time regulators (day length insensitivity and sensitivity alleles), which are also involved in developmental traits, small effects of different alleles could be masked in such studies. Hence, our study focuses on the analysis of agronomic traits in cultivars carrying day-length sensitive alleles of *Ppd-1*, with the objective of identifying novel genomic variation for fine-tuning of yield-related traits.

The previous investigations did also not include a detailed analysis of sequence polymorphisms in homoeologous *Ppd-1* gene copies. Sequence analysis of *Ppd-1* genes including the 5` upstream region in bread wheat is rare and has been performed with a low number of cultivars (Beales et al. 2007; Díaz et al. 2012; Nishida et al. 2013). Today, accurate long-read sequencing technologies provide a powerful new tool to distinguish and compare gene coding and regulatory elements for complex gene families (Xu et al. 2022; Whitford et al. 2022), even in complex polyploid crop plant genomes like that of bread wheat (Makhoul et al. 2022). This study aimed to provide a comprehensive analysis of the sequence variability of *Ppd-1* homoeologous within a diverse selection of European winter bread wheat cultivars. This was done by employing multiplex long-read sequencing methods which enabled us to survey allelic variants and dissect their influence on multiple agronomic traits, including yield components and root angle, and assess their relationship with grain yield across different environments.

## Materials and methods

### Phenotypic data

A panel of 194 bread wheat cultivars, including 190 elite European winter wheat cultivars and four cultivars from the United States and Australia, was used in this study. Two sets of phenotype data were implemented. The first dataset comprised of adjusted means for ten agronomic traits assessed across six locations and two growing seasons (2014/15, 2015/16) in field trials involving three different agrochemical treatments, designated as HiN/HiF, HiN/NoF and LoN/NoF, as described by Voss-Fels et al. (2019a). The HiN/HiF treatment received mineral fertilizer at a total nitrogen supply rate of 220 kg N ha^−1^, along with full intensity of fungicides, insecticides, and growth regulators. The HiN/NoF treatment also received a total nitrogen supply of 220 kg N ha^−1^ including N min; however, no fungicides were applied. The LoN/NoF treatment was supplied with only 110 kg N ha^−1^, and no fungicides were applied. The ten agronomic traits studied included grain yield [d*t*/ha], biomass [*t*/*h*], thousand kernel weight (TKW) [g], kernels per spike (KS), kernels per m^2^ (KSQ), spikes per m^2^ (SSQ), harvest index (HI), plant height (PH) [cm], protein yield (PY) [kg/ha], and crude protein (CP) [%] (Supplementary File 1, Table S1).

The second dataset comprised phenotypic data for seven agronomic traits related to spike morphology traits, described by Voss-Fels et al. (2019b). In this case, the phenotypic data were collected in 2016 from two different locations in Germany, Rauischholzhausen (RHH) and Gross-Gerau (GGE), under two different cropping intensities: HiN/HiF and LoN/NoF treatments. Seven traits were assessed, including days to heading, number of basal seedless spikelets per spike (BSpS), rachis length (RL), number of rachis nodes (NRN), rachis internode length (RIL = RL/NRN), spikelet fertility index (SFI = RL/NRN-BSpS) and an overall fertility index (FI = KS/RN × 4) (Supplementary File 1, Table S2). In addition, we used phenotype data for mean values of the nodal root-angle index (NRI) from three independent greenhouse experiments, as described by Voss-Fels et al. (2018) (Supplementary File 1, Table S1).

### PCR amplification, Oxford Nanopore Technologies library preparation, and sequencing of *Ppd-1* genes in 96 bread wheat cultivars

Genomic DNA was extracted from young leaf tissues using the BioSprint 96 DNA Plant kit (Qiagen, Düsseldorf, Germany) according to the manufacturer’s recommendations. DNA concentrations were estimated using Qubit dsDNA BR Assay kit from Invitrogen (Thermo Fisher Scientific, Waltham, USA) and a microplate reader with fluorescence excitation/emission (TECAN infinite F200, Männedorf, Switzerland). Two primer pairs, Ppd1ABD and Ppd1B, were designed based on a multiple alignment of previously published reference genome sequences of bread wheat (Walkowiak et al. 2020; Sato et al. 2021; IPK Crop Analysis Tools Suite 2021; Athiyannan et al. 2022). The primer pair Ppd1ABD was developed to target the coding region and promoter sequence of the three homoeologous *Ppd-1* loci on chromosomes 2A, 2B, and 2D. In contrast, the primer pair PpdB1 was designed to exclusively amplify the *Ppd-B1* locus on chromosome 2B. The target-specific primers were tailed with universal sequences at 5` end to enable the attachment of Oxford Nanopore Technologies (ONT) barcodes in a subsequent PCR reaction (primer sequences are available in Supplementary File 1, Table S3). For the first-round PCR amplification, each primer pair was separately amplified in a 25 µl reaction volume. The reaction mixture included 7 µl of nuclease-free water, 12.5 µl of GoTaq Long PCR Master Mix (Promega, Madison, WI, USA), 1.5 µl of each forward and reverse primer (10 µM), and 2.5 µl (40 ng/μl) of genomic DNA. PCR reactions were performed in a T100 Thermal Cycler (Bio-Rad Laboratories, Hercules, CA, USA) using the following program: initial denaturing at 94 °C for 2 min, followed by 35 cycles of 94 °C for 25 s, 64.5 °C annealing/extension for 8 min and 10 s, with a final extension step at 72 °C for 10 min. Agarose gel electrophoresis was used to ensure the success of PCR amplification. Subsequently, DNA quantity was measured, and PCR products obtained from amplification using two primer pairs for each cultivar’s reactions were combined in a single tube. The pooled PCR products were then purified using AMPure XP beads (Beckman Coulter, Brea, CA, USA) to remove salts, primers, small fragments, and proteins. Subsequently, the second round of PCR amplification (intended to incorporate barcode sequences into the amplicons) was carried out in 50 µl reaction volumes consisting of 25 µl GoTaq Long PCR Master Mix, 24 µl of the combined first-round PCR products, and 1 µl of a barcode primer (EXP-PBC096, ONT, Oxford, UK). PCR conditions used for barcoding were as follows: an initial denaturing step at 95 °C for 2 min, followed by 18 cycles of denaturation for 15 s at 94 °C, annealing for 15 s at 62 °C, and extension for 8 min and 10 s at 65 °C, the final extension step was carried out at 72 °C for 10 min. After PCR barcoding, the barcoded amplicons were purified using AMPure XP beads, and the quantity of PCR products for each cultivar was measured to ensure equal representation of all 96 cultivars (96 barcodes). To create a single sequencing library with all the samples, equal amounts of the barcoded amplicons from each cultivar were pooled together into a single tube. The MinION library was produced using the Ligation Sequencing Kit 1D (SQK-LSK110, ONT Oxford, UK) according to the manufacturer’s recommendations. About 30 fmol (150 ng) of pooled library was loaded and sequenced on a MinION R9.4.1 flow cell for approximately 24 h, until no further sequencing reads could be collected. After completing the run, the flow cell was washed using Flow Cell Wash Kit (EXP-WSH004, ONT, Oxford, UK) and was used again for resequencing of the same pooled library.

### Processing and analysis of Oxford Nanopore Technologies data

The raw electrical signals stored in FAST5 file format obtained from the MinION instrument were processed using the base-caller Guppy version 6.3.7 + 532d626 with model dna_r9.4.1_450bps_hac.cfg (Oxford Nanopore Technologies 2022) in a virtual machine with two NVIDIA Tesla 4 TU104GL (NVIDIA Corporation) Graphic Processor Units (GPU). The guppy_barcoder was used to demultiplex the basecalled reads, utilizing the detect_mid_strand_barcodes option to eliminate chimeric reads that were generated during both library preparation and nanopore sequencing stages. Reads with *Q*-score lower than 8 and length less than 4000 bp were filtered out by using the NanoFilt v.2.8.0 tool (De Coster et al. 2018). Filtered reads were aligned against the *Ppd-1* gene sequences of different wheat reference genomes (Walkowiak et al. 2020; Sato et al. 2021) using the NGMLR long-read mapper version 0.2.7 (Sedlazeck et al. 2018), with setting min-identity to 0.80. Subsequently, the alignment files in SAM format were converted to sorted BAM files, and only reads with a map quality score above 50 (unique mapping to single sequences) were used for downstream analyses.

### Generation of consensus sequences and variant calls from Oxford Nanopore Technologies data

Consensus *Ppd-1* sequences were generated by using a reference-guided assembly strategy where reads are mapped against the reference sequence and then used to construct a consensus sequence using Amplicon_sorter tool and Canu assembler v2.2 (Vierstraete and Braeckman 2022; Koren et al. 2017) (Supplementary File 3, 4). Polymorphisms were identified by calling variants from a multiple alignment of consensus sequences aligned to a reference sequence, considering only the regions present in the reference sequences. The SNP-sites tool was used for this analysis (Page et al. 2016). Single nucleotide polymorphisms (SNPs) with a minor allele count of less than 2 and SNPs located in homopolymeric regions were filtered out from the analysis. The identified SNPs and structural variants (SVs) were additionally subjected to visual inspection using the Integrative Genomics Viewer (Robinson et al. 2011).

### Sequencing coverage of wheat *Ppd-1* genes

Using Oxford Nanopore Technologies (ONT), PCR fragments with a total length of 11.3 kbp including both the 7850 bp 5´upstream region and the entire gene sequence of *Ppd-B1* were sequenced*.* The average sequencing coverage of *Ppd-B1* for all 96 wheat cultivars was high at 2500×. For *Ppd-A1*, approximately 8.4 kbp of PCR fragments were sequenced, covering nearly the complete gene (except for intron 7 and exon 8), along with a 5394 bp 5´ upstream region. Among 96 cultivars, five displayed either no coverage or extremely low coverage for *Ppd-A1* gene. However, the average coverage of *Ppd-A1* for 91 out of 96 cultivars was determined to be 147×.

When analyzing ONT reads from all 96 wheat cultivars, it was found that none of the reads aligned with the *Ppd-D1* gene reference which putatively results from the presence of a long stretch of AT dinucleotide repeats near the forward primer’s binding site.

### Detection and filtering chimeric reads in ONT sequencing

Despite the removal of chimeric reads resulting from library preparation and nanopores sequencing through the detection of barcodes located within the middle of reads, a significant percentage of chimeric reads, which probably originated from the PCR amplification, were still detected. In all the investigated cultivars, approximately 27% of long ONT reads (> 7 kbp) that mapped to the target *Ppd-A1* gene reference were found to be composed of distinct fragments originating from the *Ppd-A1* and from the *Ppd-B1* gene. These chimeric breakpoints or recombination events leading to the formation of the chimeric sequences occurred after a low complexity region, specifically involving poly *C* stretches as illustrated in Supplementary File 2, Fig. 1. Artificial chimeric reads can cause incorrect polymorphism calls during variant calling if not handled carefully. This was dealt with by adjusting the percentage identity threshold in mapping tools between ONT reads and the reference sequence to a value higher than 85% to improve the accuracy of mapped reads. Additionally, utilizing the integrative genomics viewer (IGV) software for visual inspection of aligned reads and sorting the ONT reads based on mismatched bases was used to detect chimeric errors and reducing incorrect variant calls. All chimeric reads were removed from subsequent analysis keeping only those that aligned perfectly with a high degree of sequence identity to the full-sequence *Ppd-A1* reference (Supplementary File 2, Fig. 1).

### Multiplex PCR assays for detecting wheat *Ppd-D1* structural variations

The variations within *Ppd-D1* gene were identified based on primers published by Beales et al. (2007) and primers derived from alignment of reference genomes. (Alignment are provided in Supplementary File 5, and primer sequences are provided in Supplementary File1, Table S4, 5.) To detect a transposable element 4819 bp in the first intron of *Ppd-D1* across 194 wheat cultivars, we developed a multiplex PCR assay consisting of three primers: a common reverse primer Ppd-D1_TransR and two forward primers Ppd-D1_TransF1 and Ppd-D1_TransF2. (Primer sequences are available in Supplementary File 1, Table S5.) PCR amplification was performed in 25 μl containing 8 μl RNase-free water, 12.5 μl of GoTaq Hot Start Green Master Mix, 1 μl of each primer (10 μM) from a total of three primers, and 1.5 μl (60 ng/μl) genomic DNA. PCR reactions were performed in a T100 thermal cycler using the following program: initial denaturing at 95 °C for 2 min, followed by 35 cycles of denaturation at 94 °C for 30 s, annealing temperature at 61 °C for 35 s, and extension at 72 °C for 1 min, with a final extension step at 72 °C for 10 min. Agarose gel electrophoresis was used to separate fragments of PCR products. Ppd-D1_TransF1 primer binds to the transposable element sequence and specifically amplifies the allele that carries the transposable element, resulting in a 532 bp PCR product. The Ppd-D1_TransF2 primer binds to intron 1 and amplifies the intact allele, producing a 329 bp PCR product (Fig. 3b). Sanger sequencing was carried out using the common reverse primer Ppd-D1_TransR to verify the specificity and accuracy of the PCR amplification. The 2089 bp deletion within the promoter region of *Ppd-D1* was identified using a primer set previously described by Beales et al. (2007).

### Development of KASP assays for polymorphisms in *Ppd-1* genes

A set of nine KASP markers was developed based on the identified polymorphisms originating from ONT sequencing and the *Ppd-1* sequences of different wheat genome references. These markers were designed to distinguish cultivars that carry different haplotype variants of *Ppd-1*. (Supplementary File 1, Table S4). The KASP procedure was conducted according to the methodology outlined in the study by Makhoul and Obermeier (2022).

### Detection of the *A. ventricosa* 2NS segment in bread wheat

To identify the wheat cultivars carrying the 2NS segment from *A. ventricosa* on the short arm of chromosome 2AS, we followed the procedure described by Helguera et al. (2003), using three PCR primers (Ventriup/LN2/Yr17neg-F). (Primer sequences are available in Supplementary File 1, Table S6.) For additional details on this protocol see https://maswheat.ucdavis.edu/protocols/Sr38.

### Statistical analyses

Statistical analysis was carried out using R software version 4.1 (R Core Team 2021). The package agricolae (De Mendiburu 2021) was used to make pairwise comparison of means for a set of groups using the Tukey honest significant difference test (Tukey HSD), with the inclusion of the argument 'unbalanced' to account for different group sizes. To estimate statistical significance for differences between two groups, parametric Student’s test and Welch’s test were applied (Student 1908; Welch 1947). Differences at *p*-value < 0.05 were considered to be significant.

## Results

### *Ppd-B1* shows a low level of polymorphisms based on Oxford Nanopore Technologies sequencing

**Fig. 1:**
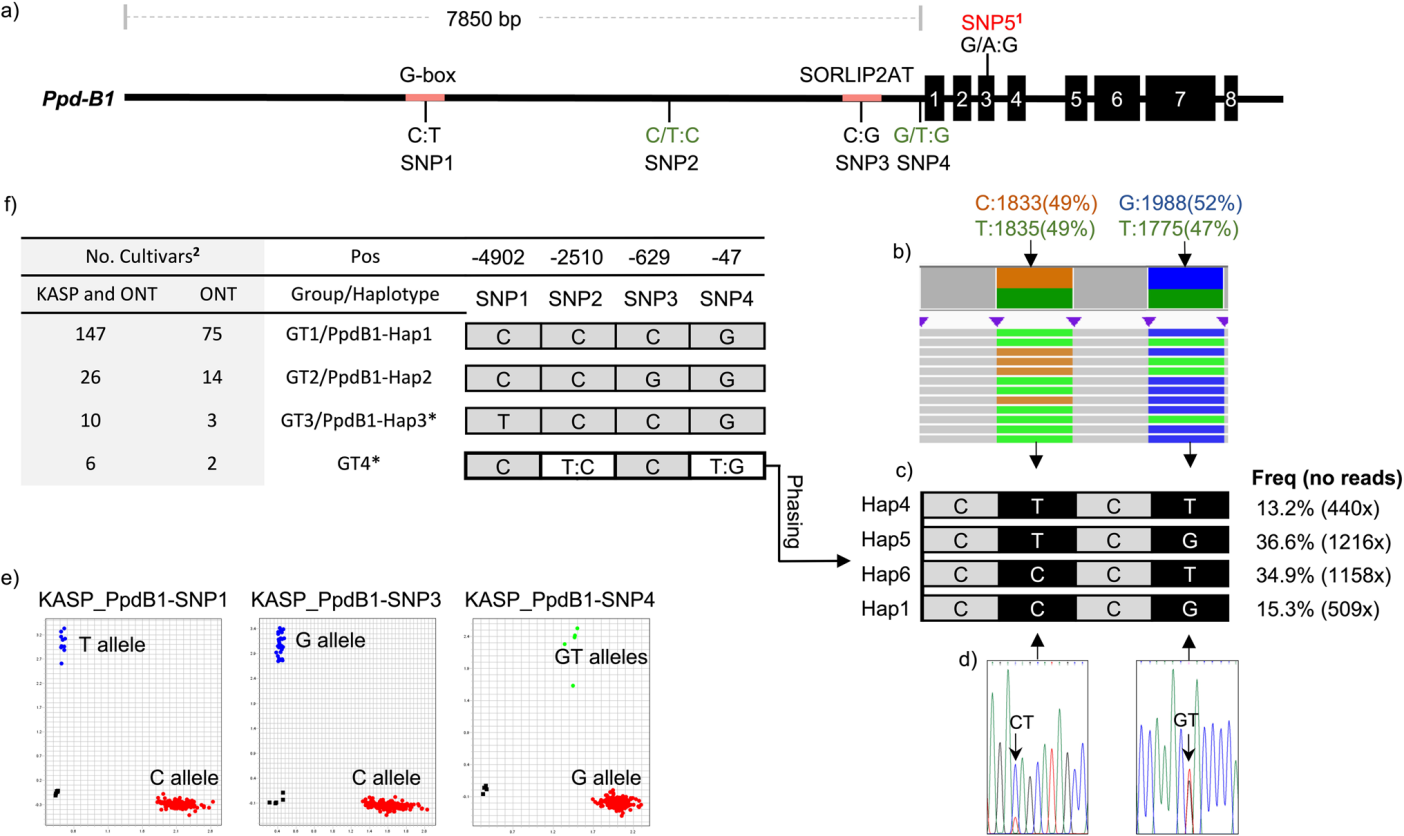
Schematic illustration of the *Ppd-B1* gene and haplotype phasing using ONT long reads. **a** Structure of the *Ppd-B1* gene, indicating the location of four SNPs in the promoter region. The black boxes represent exons. **b** IGV visualization displays the number and frequencies of ONT reads containing the alleles of the two heterozygous SNPs and their combinations in the cultivar Kormoran. **c** Four haplotypes were deduced from group GT4 using ONT long reads, and the frequency of each haplotype. **d** Two SNPs with overlapping peaks were detected in Sanger sequencing chromatograms. **e** Three genotyping plots of KASP assays used for identifying the polymorphisms of *Ppd-B1* in 189 bread wheat cultivars. **f** Groups, haplotypes, and SNPs detected in *Ppd-B1* in 189 cultivars*.* Positions of SNPs counting from the start codon. ^1^SNP5 was identified in a heterozygous state exclusively within the hybrid cultivar Hyland. ^2^ Five cultivars were not included due to missing data (Florian and Isengrain) or because they are hybrids (Hyland, Hybery and Hylux). *The GT4 and GT3 groups were excluded from the correlation analysis due to their small sample sizes

The comparison of the ONT sequences of 96 cultivars showed that *Ppd-B1* sequences were identical from the translation start codon to the 3´ UTR across almost all 96 cultivars, except for the hybrid cultivar Hyland which exhibited the presence of two alleles (A/G) for a SNP in the third exon. In contrast, within the 7850 bp 5´upstream region of the gene four SNPs were detected (Fig. 1a). Two of these SNPs (SNP2 and SNP4) located at positions − 47 bp and − 2510 bp upstream of the start codon, respectively, were found to be in a heterozygous state in two wheat cultivars, Sperber and Kormoran. Previous work conducted by Langer et al. (2014) and Würschum et al. (2015) had reported that these two cultivars harbor two copies of the *Ppd-B1* gene using the TaqMan assay qPCR method. The method applied in our study based on the extraction of long read sequences unveiled the presence of four distinct haplotypes in these two cultivars, each supported by an approximate depth of reads over 350× (Fig. 1b, c). This could either be due to the presence of four copies or the presence of two copies in a heterozygous state of the *Ppd-B1* gene in both cultivars, Sperber and Kormoran.

Analysis of the cis-regulatory elements in the promoter region of *Ppd-B1*, using the PlantCARE (Lescot et al. 2002) and PLACE databases (Higo et al. 1999), revealed that SNP1 and SNP3 are located within the G-box sequence and SORLIP2AT sequence (Sequences Over-Represented in Light-Induced Promoters), respectively. The G-box and SORLIP elements play crucial roles in regulating gene expression in response to light and other environmental signals in plants (Hudson and Quail 2003; Kim et al. 2003; Schindler et al. 1992; Yadav et al. 2005). However, these two SNPs were detected by previous studies showing no significant association with photoperiod insensitivity in bread wheat (Beales et al. 2007; Díaz et al. 2012; Kiseleva et al. 2017). Based on the identification of four SNPs, the 93 cultivars were classified into four groups and six haplotypes. Among the four groups, GT1, which includes the PpdB1-Hap1 haplotype, represented the majority of cultivars, accounting for 80% of the cultivars. The remaining groups, GT2 and GT3, consisted of the PpdB1-Hap2 and PpdB1-Hap3 haplotypes, respectively, making up 15% and 3.2% of the cultivars. Additionally, two cultivars carrying heterozygous SNPs in their sequences (four haplotypes of *Ppd-B1*) were assigned to group GT4. To enhance statistical comparisons of *Ppd-B1* variants, we developed three KASP assays capable of detecting alleles at SNP1, SNP3, and SNP4. Subsequently, these KASP markers along with Sanger sequencing were used to genotype an additional panel of 98 bread wheat cultivars (Fig. 1d, e), resulting in an expanded number of cultivars identified within each genotype group as illustrated in Fig. 1f.

### *Ppd-A1* shows novel structural variations and a high number of SNPs based on Oxford Nanopore Technologies sequencing

**Fig. 2:**
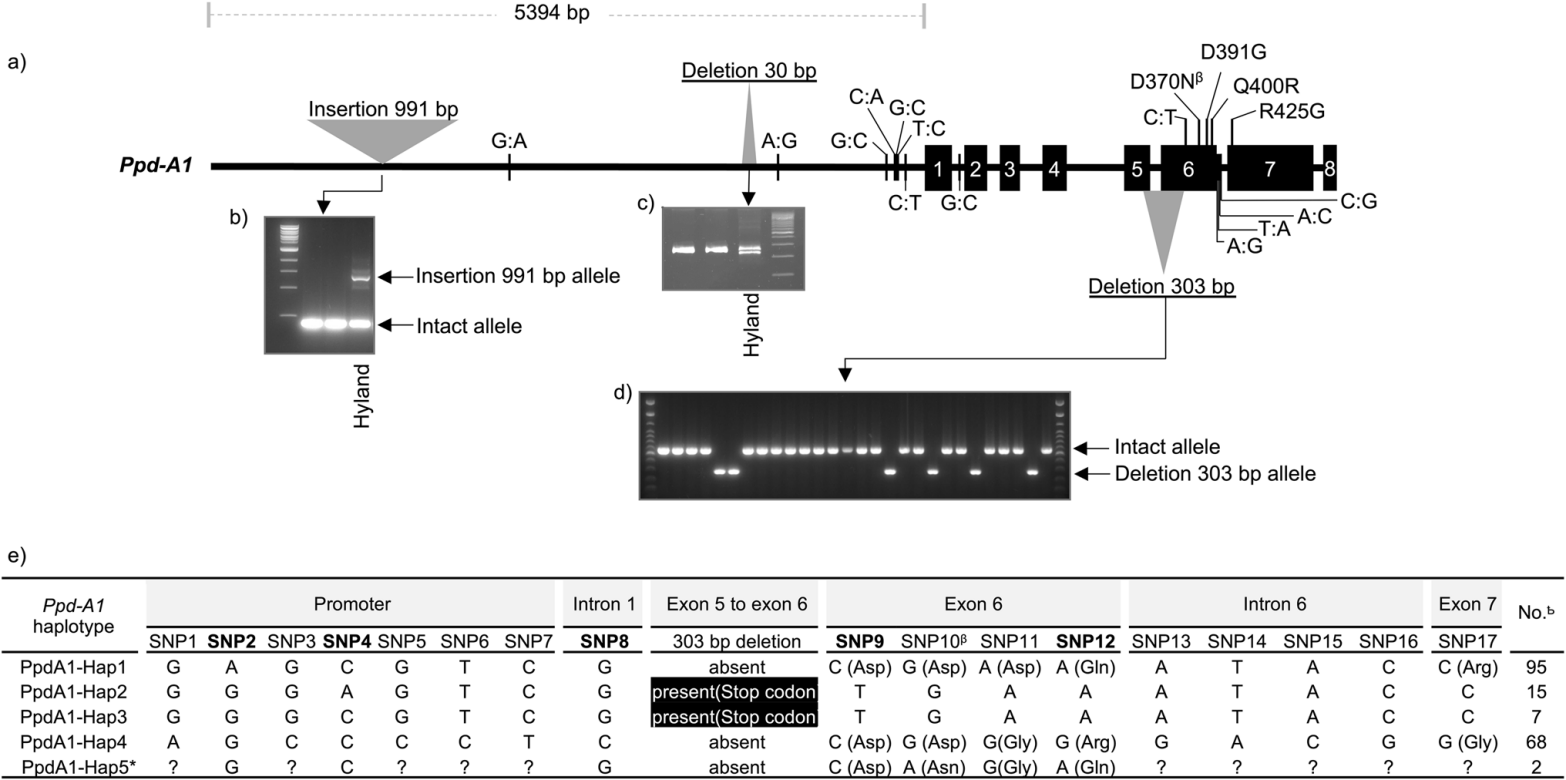
Variations in the *Ppd-A1* gene and its promoter. **a** Structure of the *Ppd-A1* gene illustrating all polymorphisms detected by ONT sequencing. The numbers in black boxes represent exons. **b**, **c**, Gel electrophoresis images displaying PCR products containing the 991 bp insertion and 30 bp deletion in the hybrid Hyland cultivar, respectively. **d** Gel electrophoresis image showing PCR product carrying the 303 bp deletion. **e** Haplotype variants identified in 187 wheat cultivars. *PpdA1-Hap5 haplotype was identified using KASP assays and Sanger sequencing. The question marks within the PpdA1-Hap5 haplotype indicate missing data. ^β^Allele A for SNP10 was detected by Sanger sequencing in two cultivars specifically associated with the PpdA1-Hap5 haplotype. The five SNPs highlighted in bold font correspond to the KASP assays designed for these specific SNPs. ^Ҍ^ Seven cultivars were not included due to missing data (Premio, Isengrain, Boregar and Soissons) or because they are hybrid cultivars (Hyland, Hybery and Hylux). The PpdA1-Hap5 haplotype was excluded from the correlation analysis due to its small sample size

Analysis of the ONT data revealed two novel structural variations, a large insertion of 991 bp and a deletion of 30 bp starting at positions − 4112 bp and − 1431 bp upstream of the start codon, respectively. Both SV alleles were present alongside the intact alleles in the hybrid cultivar Hyland (Fig. 2a–c). Additionally, a 303 bp deletion spanning from the middle of exon 5 to the middle of exon 6 was identified in 13 wheat cultivars (Fig. 2d). This deletion is predicted to result in a premature stop codon and a truncated protein lacking the CCT domain (Beales et al. 2007; Shaw et al. 2012). The analysis of SNPs from the ONT data unveiled a total of 16 SNPs within the *Ppd-A1* sequence. Among these, seven SNPs were identified upstream of the start codon. Furthermore, within exon 6 and exon 7, four SNPs were detected, of which three led to predicted amino acid substitutions: D391G, Q400R and R425G. We developed two PCR assays and five KASP markers for detecting these SVs and SNPs in an additional panel comprising 98 of bread wheat cultivars. (Primer sequences are provided in Supplementary File1, Table S4 and S5.) After genotyping the complete panel using the PCR and KASP assays, as well as Sanger sequencing, the 187 cultivars can be classified into five haplotype variants, PpdA1-Hap1 and PpdA1-Hap4 were the most prominent in the panel at frequencies of 51% and 36%, respectively. PpdA1-Hap2 and Hap3, on the other hand, differ from each other with only one SNP in the promoter region, representing 8% and 4% of the cultivars, respectively, while PpdA1-Hap5, which was not detected in the samples sequenced by ONT, was specifically present in two cultivars, Kobold and Renesansa (Fig. 2e).

### The photoperiod insensitive *Ppd-D1a* allele (PpdD1-Hap3) is associated with root growth and grain yield components

**Fig. 3:**
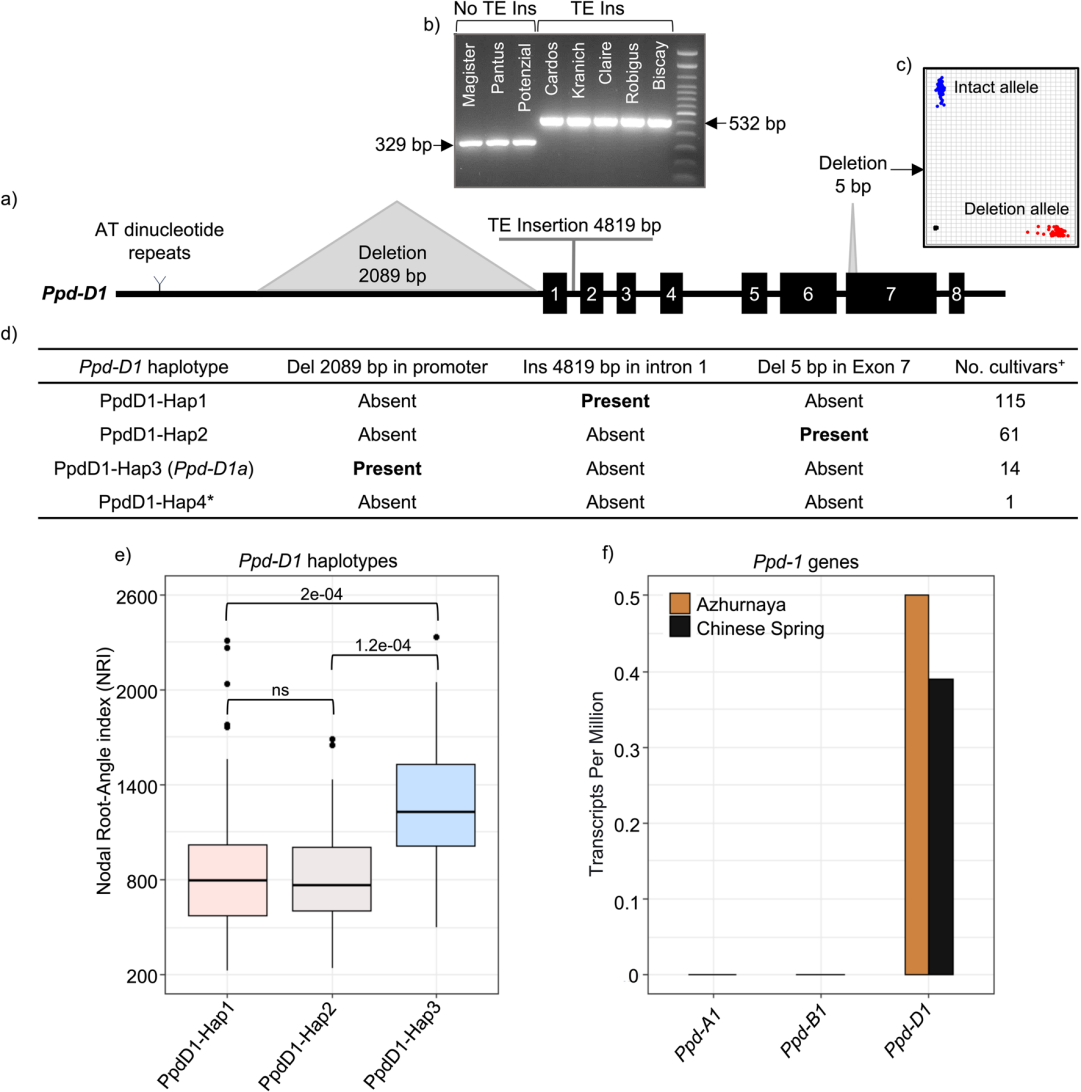
Schematic representation of the *Ppd-D1* gene structure, polymorphisms, and correlation with nodal root angle index trait. **a** Wheat *Ppd-D1* gene with black boxes indicating exons and highlighting three structural variations. **b** PCR products obtained using multiplex primers to identify the cultivars with the transposable element in the first intron. **c** Genotyping plot of KASP assay used to detect cultivars carrying the 5 bp deletion in exon 7. **d** Four haplotypes of *Ppd-D1* detected in 191 wheat cultivars. **e** Pairwise comparisons between three haplotypes of the *Ppd-D1* gene and the NRI trait, with the corresponding *p*-value. **f** Expression patterns of three homoeologous *Ppd-1* genes in the root were examined in two wheat cultivars Azhurnaya and Chinese Spring using RNA-seq data downloaded from the Wheat Expression Browser www.wheat-expression.com. ^+^Three cultivars were excluded from analyses because they are hybrids (Hyland, Hybery and Hylux). *The haplotype PpdD1-Hap4 was omitted from the correlation analysis due to its small sample size

*Ppd-D1* gene is classified into four haplotypes based on three well-known polymorphisms: a 2089 bp deletion in the promoter, a 4819 bp insertion in the first intron, and a five bp deletion in exon 7 (Beales et al. 2007; Guo et al. 2010), as illustrated in Fig. 3a–d. Our findings showed that cultivars carrying the PpdD1-Hap3 haplotype (insensitive *Ppd-D1a* allele) exhibited a significantly earlier heading by five to seven days in both treatments at GGE and RHH locations compared to cultivars carrying the other haplotypes (*p*-value < 0.00001). Furthermore, cultivars carrying insensitive *Ppd-D1a* allele showed a significant reduction in biomass, PY, PH, NRN, KS, TKW, and number of BSpS in most environments. We did not find a statistically significant difference in grain yield when comparing between wheat cultivars with the insensitive *Ppd-D1a* allele and those with sensitive allele. (Supplementary File 2, Fig. 2).

Most surprisingly, the cultivars carrying PpdD1-Hap3 showed a significantly increased nodal root-angle index. This increase indicated that these cultivars had narrower and deeper roots, with a remarkable 57% increase compared to cultivars carrying the other two haplotypes (Fig. 3e). To further investigate the effect of *Ppd-D1* expression on root growth, we accessed publicly available RNA-Seq data from the Wheat Expression Browser database (Ramírez-González et al. 2018). This analysis was conducted across various tissues, including root, leaf/shoot, spike, and grain, for the wheat cultivars Azhurnaya and Chinese Spring. Our analysis of the publicly accessible expression data revealed that from the three homoeologous genes only the *Ppd-D1* gene showed transcript expression in root tissue (Fig. 3f). This finding provides additional support for the putative involvement of *Ppd-D1* in root architecture.

In our investigation, we found two widespread mutations in *Ppd-D1*: the 4819 bp mariner-type transposon insertion and a 5 bp deletion in exon 7, referred to as PpdD1-Hap1 and PpdD1-Hap2, respectively. To gain a deeper understanding of how these mutations associate with agronomic traits, we conducted a comparative analysis involving two distinct groups. Group 1 comprised a total of 68 cultivars characterized by the combination of PpdA1-Hap1 plus PpdD1-Hap1. Similarly, group two consisted of 21 cultivars carrying the combination PpdA1-Hap1 plus PpdD1*-*Hap2. Both groups exhibited a single copy of *Ppd-B1*. Our analysis revealed no significant and environmentally consistent differences between the two mutations across 17 tested traits (Supplementary File 2, Fig. 3).

### *Ppd-B1* haplotypes are associated with spike traits under low cropping intensities

The effect of the two highly frequent haplotypes, PpdB1-Hap1 (GT1) and PpdB1-Hap2 (GT2), which differ by a single SNP, on agronomic traits, was investigated by comparing two distinct groups. Group 1 comprised a total of 38 cultivars characterized by the combination of PpdA1-Hap4 plus PpdB1-Hap1 (GT1), while group two consisted of 21 cultivars carrying the combination PpdA1-Hap4 plus PpdB1-Hap2 (GT2). We excluded the cultivars carrying the insensitive *Ppd-D1a* allele (PpdD1-Hap3) from this analysis. Significant differences between PpdB1-Hap1 (GT1) and PpdB1-Hap2 (GT2) were observed for four traits correlated with spike architecture, but only under low cropping intensities, the LoN/NoF treatment (110 kg N ha^−1^ and no fungicides) at both the RHH and GGE locations. PpdB1-Hap2 (GT2) was significantly associated with a decrease in RIL, SFI, FI, and an increase in BSpS (for more information, see Supplementary File 2, Fig. 4).

### *Ppd-A1* haplotypes are associated with multiple agronomic traits

**Fig. 4:**
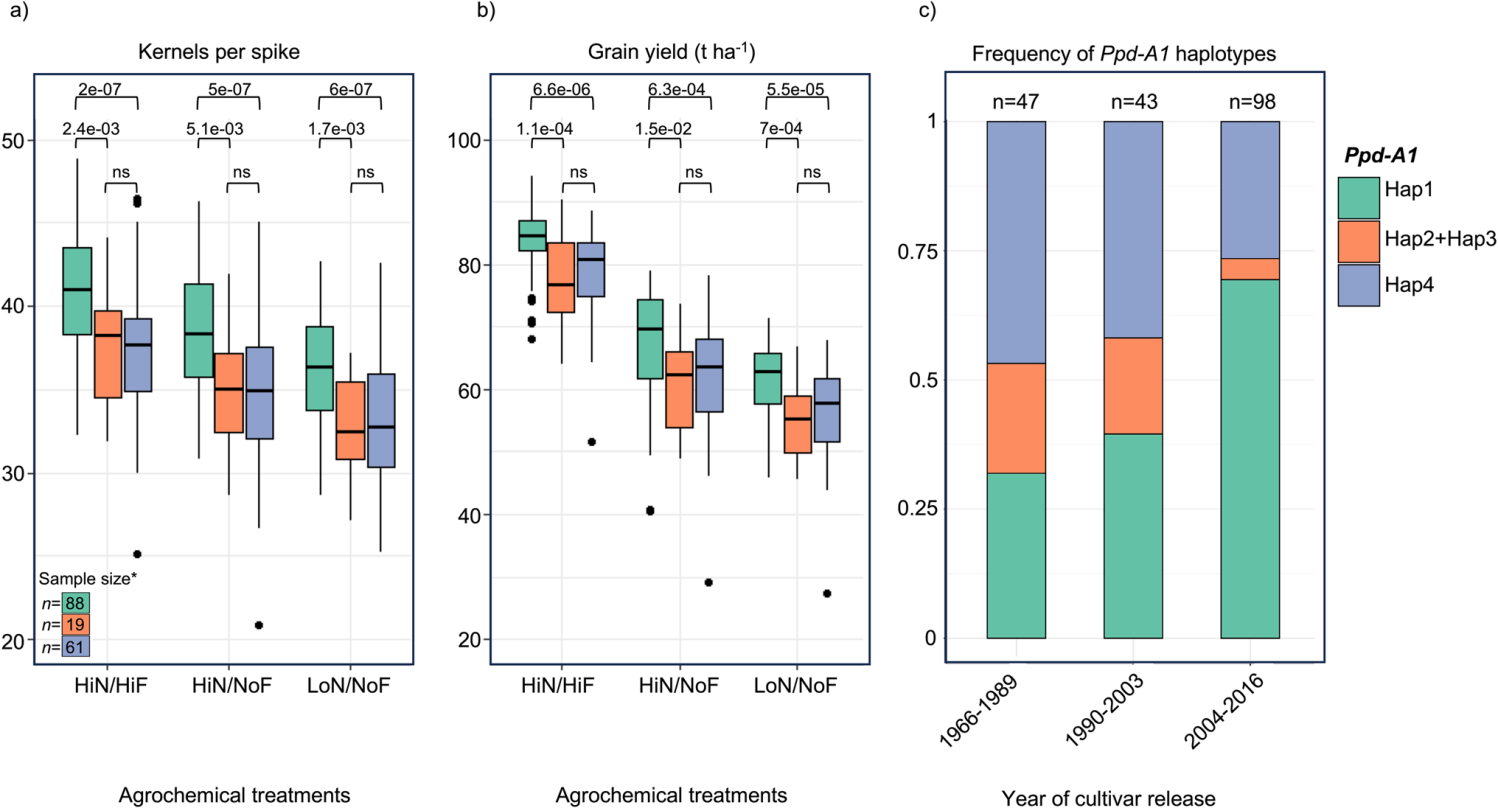
Analysis of *Ppd-A1* haplotypes and their associations with agronomic traits in winter bread wheat cultivars. **a**, **b** Pairwise comparisons of different haplotypes of the *Ppd-A1* gene with kernels per spike and grain yield, with the corresponding *p*-values. The experiment includes three agrochemical treatments: high nitrogen with fungicide (HiN/HiF), high nitrogen without fungicide (HiN/NoF), and low nitrogen without fungicide (LoN/NoF). * Cultivars carrying the insensitive *Ppd-D1a* allele (PpdD1-Hap3) or multiple copy number of *Ppd-B1* (PpdB1-GT4) were excluded from this analysis. **c** Frequency distribution of *Ppd-A1* gene haplotypes among 188 winter bread wheat cultivars released over a period of 50 years of breeding. The number above each bar indicates the count of cultivars for each time group

To explore the influence of *Ppd-A1* haplotype differences on agronomic traits, a statistical comparison was conducted between two groups comprising the most frequent haplotypes, PpdA1-Hap1 and PpdA1-Hap4. These two haplotypes can be distinguished by a total of 14 SNPs, with three of them being non-synonymous SNPs located in exon 6 and exon 7. The first group consisted of 88 cultivars carrying the potentially functional PpdA1-Hap1 haplotype, while the second group included 61 cultivars harboring the potentially functional PpdA1-Hap4 haplotype. The cultivars carrying the insensitive *Ppd-D1a* allele (PpdD1-Hap3) or multiple copy number of *Ppd-B1* (PpdB1-GT4) were excluded from this analysis. The association study revealed that the haplotype PpdA1-Hap1 was significantly associated with increased grain yield, biomass, KS, HI and KSQ and with decreased PH and CP compared to the haplotype PpdA1-Hap4 across all tested environments (Supplementary File 2, Fig. 5). The average increase was very high with 10% and 8% for KS and grain yield, respectively (Fig. 4a, b). Moreover, the PpdA1-Hap1 haplotype showed a significant and positive correlation with SFI and FI when compared to haplotype PpdA1-Hap4 across all studied environments, resulting in an average increase of 9.1% and 11.2%, respectively. We observed that cultivars carrying a 303 bp deletion spanning from exon 5 to exon 6 (referred to as PpdA1-Hap2 and PpdA1-Hap3) showed no significant differences in grain yield, KS, KSQ, biomass, CP, and HI when compared to cultivars harboring the haplotype PpdA1-Hap4 across all tested environments.

### Change of *Ppd-1* allele frequencies across 5 decades of breeding

To investigate the selection process of *Ppd-1* haplotypes in wheat breeding history, we analyzed the frequency of *Ppd-A1*, *Ppd-B1* and *Ppd-D1* haplotypes in 192 elite wheat cultivars released between 1966 and 2016 (Supplementary File 1, Table S7). Over the course of 50 years of wheat breeding, the frequency of haplotype PpdA1-Hap1 showed an increase, rising from 30 to 70% (Fig. 4c). In contrast, the frequencies of haplotype PpdA1-Hap4 and both haplotypes PpdA1-Hap2/Hap3 decreased from 47 to 26% and from 21 to 4.7%, respectively. This change in haplotype frequencies was quite different for *Ppd-D1* and *Ppd-B1*. For both of these genes, a high frequency of haplotype 1 was maintained from 1966 to 2016 (Supplementary File 2, Fig. 6).

### Identification of the 2NS segment from *A. ventricosa* and its influence on grain yield and other traits in European wheat cultivars

We detected the presence of the 2NS/2AS translocation in our panel of wheat cultivars using a gel-based PCR method following the protocol outlined by Helguera et al. (2003). Our analysis identified 51 cultivars with the 2NS/2AS translocation, of which 48 exhibit the PpdA1-Hap1 haplotype. In contrast, the remaining cultivars without the 2NS/2AS translocation exhibit a diverse distribution of *Ppd-A1* haplotypes, with 46 carrying PpdA1-Hap1, 65 having PpdA1-Hap4, 15 carrying PpdA1-Hap2, seven displaying PpdA1-Hap3, and two possessing PpdA1-Hap5 (Supplementary File 1, Table S7). We observed that out of the 94 cultivars carrying the PpdA1-Hap1 haplotype, 48 (51%) also possessed the 2NS/2AS translocation. To assess the impact of the 2NS/2AS translocation on agronomic traits and dissect it from the PpdA1-Hap1 effect, we divided the cultivars carrying the PpdA1-Hap1 haplotype into two groups based on the presence or absence of the 2NS/2AS translocation. The results of the association study revealed a significant correlation between the 2NS/2AS translocation and many agronomic traits (Table 1, Supplementary File 1, S8). The presence of the 2NS/2AS translocation was associated with significantly higher grain yield, compared to those haplotypes without the translocation, with an increase 5–17% depending on the treatment. However, PpdA1-Hap1 with 2NS/2AS translocation showed no significant difference for SFI, FI, PH and CP compared to PpdA1-Hap1 without translocation under most environments. Moreover, no significant differences were observed between these two groups for HI, KS, KSQ traits under high cropping intensity (HiN/HiF treatment), as indicated in Table 1. This translocation has also been described to be involved in multiple disease resistance (Bariana and McIntosh 1993; Agarwal et al. 2021). We confirmed that the translocation is associated with increased resistance against powdery mildew and stripe rust, but in cultivars without translocation no association of PpdA1-Hap1 or PpdA1-Hap4 with resistance against these two pathogens was detected (Supplementary File 1, S9).

**Table 1:** Comparison of the means of different agronomic traits between three groups displaying 2NS/2AS translocation and distinct *Ppd-A1* haplotypes

Traits	Location^1^	Treatment^2^	With 2NS/2AS translocation	No 2NS/2AS translocation	
			PpdA1-Hap1	PpdA1-Hap1	PpdA1-Hap4
			Mean (*n* = 46)^3^	Mean (*n* = 41)^3^	Mean (*n* = 58)^3^
Grain yield [dt/ha]	A6-2	HiN/HiF	85.31^a^	81.8^b^	78.45^c^
		HiN/NoF	72.33^a^	61.7^b^	61.32^b^
		LoN/NoF	64.51^a^	57.71^b^	56.26^b^
Harvest index	A6-2	HiN/HiF	0.49^a^	0.48^ab^	0.46^b^
		HiN/NoF	0.46^a^	0.44^b^	0.43^b^
		LoN/NoF	0.44^a^	0.43^b^	0.42^b^
Kernels per spike	A6-2	HiN/HiF	41.62^a^	40.3^a^	37.14^b^
		HiN/NoF	39.57^a^	36.8^b^	34.5^c^
		LoN/NoF	37.23^a^	35.3^b^	32.8^c^
Kernels per m^2^	A6-2	HiN/HiF	21,329.9^a^	20,570.3^a^	18,740.1^b^
		HiN/NoF	19,658.1^a^	17680^b^	16,685.3^b^
		LoN/NoF	17,089.3^a^	15861^b^	14,798.3^c^
Thousand Kernel Weight [g]	A6-2	HiN/HiF	43.06^a^	42.67^a^	44^a^
		HiN/NoF	39.74^a^	38.10^b^	40.13^a^
		LoN/NoF	42.06^a^	40.84^a^	42.18^a^
Protein yield [kg/ha]	A6-2	HiN/HiF	1102.9^a^	1071.36^b^	1047.87^b^
		HiN/NoF	919.24^a^	790.42^b^	796.17^b^
		LoN/NoF	679.32^a^	618.7^b^	615.13^b^
Biomass [t/h]	A6-2	HiN/HiF	1780.7^a^	1737.8^b^	1703.8^b^
		HiN/NoF	1604.9^a^	1458.1^b^	1477.7^b^
		LoN/NoF	1496.3^a^	1396.9^b^	1399.5^b^
Plant height [cm]	A6-2	HiN/HiF	84.38^b^	85.5^b^	91.4^a^
		HiN/NoF	85.31^b^	86.1^b^	92.1^a^
		LoN/NoF	88.46^b^	90.34^b^	97.12^a^
Crude protein [%]	A6-2	HiN/HiF	13.01^b^	13.2^b^	13.5^a^
		HiN/NoF	12.81^b^	12.9^ab^	13.2^a^
		LoN/NoF	10.58^c^	10.84^b^	11.13^a^
Spikelet fertility index	RHH	HiN/HiF	2.52^a^	2.5^a^	2.33^b^
		LoN/NoF	2.28^a^	2.24^a^	2.05^b^
	GGE	HiN/HiF	2.56^a^	2.42^a^	2.25^b^
		LoN/NoF	2.35^a^	2.24^a^	2.08^b^
Overall fertility index	RHH	HiN/HiF	0.55^a^	0.54^a^	0.50^b^
		LoN/NoF	0.49^a^	0.47^a^	0.43^b^
	GGE	HiN/HiF	0.59^a^	0.54^ab^	0.50^b^
		LoN/NoF	0.52^a^	0.50^ab^	0.46^b^

*The cultivars carrying the insensitive *Ppd-D1a* allele (PpdD1-Hap3) or multiple copy number of *Ppd-B1* (PpdB1-GT4) were excluded from this analysis

^1^A6-2 indicates an adjusted means across six locations and two growing seasons. The RHH and GGE represent Rauischholzhausen and Gross-Gerau locations, respectively

^2^The experiment includes three agrochemical treatments: high nitrogen with fungicide (HiN/HiF), high nitrogen without fungicide (HiN/NoF), and low nitrogen without fungicide (LoN/NoF)

^3^Means followed by different letters are significant differences among haplotypes according to post hoc Tukey HSD test (*p* < 0.05)

## Discussion

### Photoperiod insensitivity alleles for *Ppd-A1* and *Ppd-B1* are not common in European wheat

Our analysis of a 7850 bp region 5′ upstream of the *Ppd-B1* gene did not identify any structural variants in this region in the analyzed predominantly European winter wheat cultivars. This is consistent with other studies which did not find *Ppd-B1* photoperiod insensitivity alleles in hexaploid wheat landraces and varieties. The same has been reported for *Ppd-A1* photoperiod insensitivity alleles in bread wheat (Bentley et al. 2011, 2013; Langer et al. 2014). However, we found a novel 991 bp insertion and 30 bp deletion in the 5′ upstream region of the *Ppd-A1* gene. Since these variations occur within a region known to influence gene expression (Wilhelm et al. 2009; Shaw et al. 2012; Nishida et al. 2013), we hypothesize that these variations may affect photoperiod response. The discovery of these new structural variations in the promoter region of the *Ppd-A1* gene is important, as plant breeders can target them in plant breeding programs.

### The *Ppd-D1a* photoperiod insensitivity allele is associated with nodal root-angle trait

Variations in the *Ppd-1* photoperiod genes have long been recognized as major-effect genes that control both flowering time and above-ground shoot growth (González et al. 2005; Beales et al. 2007; Boden et al. 2015; Royo et al. 2016, 2018; Steinfort et al. 2017a, b; Ramirez et al. 2018; Arjona et al. 2018; Pérez-Gianmarco et al. 2019; Zhang et al. 2019; Wu et al. 2021). In this study, we observed that the photoperiod insensitivity allele *Ppd-D1a* (PpdD1-Hap3) had the most significant impact on the nodal root-angle index, showing a remarkable 57% increase compared to considerably lower effects on other traits (e.g., − 10% for biomass, − 6% for NRN). The connection between flowering genes and root development has been investigated in a few previous studies. Lorenzo et al. (2023) found that overexpressing the *TERMINAL FLOWERING 1* (MsTFL1A) gene in alfalfa (*Medicago sativa*) consistently resulted in delayed flowering, alterations in inflorescence architecture, and reduced root development, suggesting that MsTFL1A serves not only as a flowering repressor but also as a regulator of root development. Bouché et al. (2016) demonstrated that flowering genes in the roots of *Arabidopsis thaliana* exhibit differential expression patterns during flowering, concluding that roots may play a role in flowering. They also observed significant variations in the root transcriptome when plants were exposed to photoperiodic treatments inducing flowering. Moreover, previous studies have shown that novel molecular variants of *VERNALIZATION* gene in winter wheat germplasm (Voss-Fels et al. 2018) and spring barley collection (Abdel-Ghani et al. 2019) are responsible for modulating root development. The *Ppd-D1a* allele, found in 14 cultivars, shows a strong correlation with both root growth angle and early flowering. This implies that utilizing this allele in breeding programs for winter wheat could be advantageous. It may lead to the development of cultivars that not only flower earlier but also have a deeper root system, facilitating improved access to soil moisture and helping to alleviate drought stress.

### Co-inheritance of the *A. ventricosa* 2NS segment and PpdA-Hap1 haplotype in wheat

We conducted Oxford Nanopore long read sequencing to analyze the *Ppd-A1* gene variability across predominantly European winter wheat cultivars, covering 8.4 kbp of the *Ppd-A1* gene and its promoter region. This approach led to the discovery of an unexpectedly high number of polymorphisms in both the promoter and exons of the *Ppd-A1* gene sequence, confirmed by Sanger sequencing and/or KASP markers. High variations within the *Ppd-A1* gene were previously documented by Takenaka and Kawahara (2012), who identified 67 haplotypes within the *Ppd-A1* gene in a panel of 158 accessions of tetraploid emmer wheat landraces. Additionally, Muterko et al. (2015) unveiled 13 haplotypes based on partial promoter sequences of 452 bp amplicons found in various wheat species. The extensive SNP diversity observed within the *Ppd-A1* gene in our panel, in comparison with the other two homoeologous genes, indicates substantial evolutionary changes in the *Ppd-A1* gene on chromosome 2A among European cultivars over the past 5 decades. Previous studies have reported the introduction of the *A. ventricosa* 2NS segment into the telomere of the short arm of wheat chromosome 2A as part of breeding programs aimed at transferring genes related to pathogen resistance (McIntosh et al. 1995; Jahier et al. 2001; Fang et al. 2011; Williamson et al. 2013; Cruz et al. 2016; Xue et al. 2018; Kishii 2019). Recently, Walkowiak et al. (2020) identified the boundaries of this translocation, spanning approximately position 0–33 Mb in the telomeric region of the short arm of the 2A chromosome using the reference wheat cultivar Jagger. Our investigation revealed that the *Ppd-A1* gene resides approximately 13 Mb outside the boundaries of this translocated region. In this study, 28% of our cultivars were found to carry the *A. ventricosa* 2NS segment. The French cultivar Apache and the German cultivar Cardos, released in 1998, were the first ones in our wheat panel to harbor this translocation. This finding is consistent with previous research by Dadshani et al. (2021) and Gao et al. (2021) who demonstrated that the incorporation of 2NS/2AS translocation from *A. ventricosa* into wheat breeding programs began in the early 1990s.

Furthermore, we observed that 94% of cultivars carrying the 2NS/2AS translocation also exhibited the PpdA1-Hap1 haplotype on chromosome 2A. This haplotype was identified in 20 wheat cultivars released between 1966 and 1996, indicating the PpdA1-Hap1 was already present in wheat cultivars prior to the introgression of the *A. ventricosa* 2NS segment into wheat. Taking these findings into account, we propose that the original parent wheat cultivar VPM-1, which was the first cultivar used to introduce 2N segment from *A. ventricosa* (Doussinault et al. 1983), probably possessed the PpdA1-Hap1 haplotype. This suggests that a large DNA segment, encompassing both PpdA1-Hap1 and the 2NS translocation, was inherited and passed on through crossing in subsequent plant breeding programs, with limited recombination occurring between the translocation and the *Ppd-A1* gene. This finding agrees with Gao et al. (2021), who observed that the region following the *A. ventricosa* segment has low polymorphism levels among the four wheat cultivars carrying the 2NS introgression (Mace, CDC Stanley, Jagger and Mattis).

We found that the cultivars carrying both the PpdA1-Hap1 haplotype and the *A. ventricosa* 2NS segment are significantly associated with increased grain yield, conferring an average 8% yield advantage across all environments. This result leads us to propose that the significant enhancement in performance observed in cultivars carrying the PpdA1-Hap1 haplotype may be partly attributed to the presence of the 2NS/2AS translocation and not only to polymorphism of *Ppd-A1* gene. Our study revealed that under high cropping intensity, PpdA1-Hap1, even without the 2NS/2AS translocation was significantly linked to increased grain yield, KS and KSQ compared to PpdA1-Hap4 haplotype, conferring a 4% yield advantage. Furthermore, the presence of PpdA1-Hap1, even in the absence of 2NS/2AS, was associated with increased SFI and KS compared to PpdA1-Hap4 across different environments. The strong correlation between kernel number and spike fertility index among wheat cultivars was previously demonstrated by González et al. (2011). The short arm of chromosome 2A, where *Ppd-A1* is located, is a hot spot for genes which have minor effects on multiple yield-related traits. Several previous genome-wide association studies have identified specific regions on chromosome 2A associated with quantitative trait loci (QTL) that influence both physiological and agronomic traits in wheat (Guo et al. 2017; Shi et al. 2017; Wolde et al. 2019; Gahlaut et al. 2019; Hu et al. 2020; Kaur et al. 2020; Pshenichnikova et al. 2021; Ding et al. 2022). Our analysis is based on the correlation of variants of *Ppd-1* with agronomic traits without controlling for population structure in a set of cultivars with diverse genomic backgrounds. Further investigations are required to confirm the causal relationship between *Ppd-1* or other tightly linked gene variations and these agronomic traits.

### *Ppd-1* haplotypes underwent positive selection across a timeline spanning 5 decades of release of European wheat cultivars

Under our growing environments in central Germany, 14 cultivars carrying haplotype PpdD1-Hap3 (*Ppd-D1a*) from which eight originated from Central Europe (Serbia, Slovenia, Moldova and Czech Republic) showed a significant reduction in some yield components like KS, NRN, biomass, PY and TKW in most environments. The insensitive *Ppd-D1a* allele has not been favored by plant breeders in North European breeding programs due to lower performance compared to sensitive alleles (Worland 1996; Cockram et al. 2007; Guo et al. 2010; Bentley et al. 2011; Langer et al. 2014). Consequently, the notably low frequency of *Ppd-D1a* observed in the analyzed panel containing predominantly cultivars released in Northern Europe can be attributed to this selective pressure.

The two haplotypes PpdD1-Hap1 and PpdD1-Hap2 have been associated with the photoperiod sensitivity phenotype by Beales et al. (2007) and Shaw et al. (2012, 2013). In contrast, Guo et al. (2010) reported that the PpdD1-Hap2 haplotype (characterized by a 5 bp deletion in exon 7) was associated with an intermediate heading date at two Chinese locations with differing day length. However, we did not find any significant differences in heading time or other agronomic traits between these two haplotypes under central German long day growing conditions. This neutral phenotypic effect of these two haplotypes observed in German conditions could possibly be due to the predicted loss or weakening of protein functionality (Beales et al. 2007; Shaw et al. 2013, 2020; Bentley et al. 2013).

The allele frequency ratio of PpdD1-Hap1 to PpdD1-Hap2 during 50 years of breeding stayed similar, suggesting that neither haplotype has been subjected to significant selective pressures. This lack of selection for or against either haplotype further supports the hypothesis of a neutral phenotypic effect for both PpdD1-Hap1 and PpdD1-Hap2 under German growing conditions. The same scenario was noted for *Ppd-B1* gene. Also, the ratio of PpdB1-Hap1 and PpdB1-Hap2 alleles remained relatively constant across the 5 decades. PpdB1-Hap1 was associated with improvements in certain yield trait components, such as RIL, SFI, FI and BSpS, but only under low cropping intensities. However, there was no significant difference in grain yield compared to PpdB1-Hap2 across all environments.

In contrast, we found a significant shift in the frequency of *Ppd-A1* haplotypes over a 50-year breeding period, leading to a remarkable prevalence of the PpdA1-Hap1 haplotype in modern cultivars compared to older ones. This strongly suggests that breeding selection has favored and enriched this specific haplotype, which is associated with improved grain yield and other agronomic traits in modern European wheat cultivars.

The strong frequency increase of the PpdA1-Hap1 allele in our panel occurred after 2004. This coincides with the introduction of the strongly linked 2NS/2AS translocation into wheat breeding programs in the beginning of the 1990s and subsequent extensive uses by CIMMYT and US breeding programs (Gao et al. 2021). Another possible reason for the strong shift in the ratio of the most common *Ppd-A1* alleles, but not for the most common *Ppd-B1* and *Ppd-D1* alleles, might be that in early stages of bread wheat breeding, plant breeders focused on selecting either insensitive or sensitive photoperiod alleles with major effects on yield under specific climatic conditions in Europe. They gave a particular attention to the *Ppd-D1* and *Ppd-B1* genes, which are known for their polymorphisms that significantly influence flowering time (Worland et al. 1998; Langer et al. 2014; Kiss et al. 2014; Bloomfield et al. 2018, 2023). Contrary to this, the *Ppd-A1* gene has no impact on flowering time and has a minor effect on yield, thus, not attracting attention from plant breeders to select for flowering time modulation early on between 1966 and 2003.

## Conclusion

Our findings reveal that targeted long-read sequencing can help detect novel polymorphisms in well-known genes. The discovery of novel structural variations and high number of SNPs in the *Ppd-1* region is providing valuable resources for utilization in marker-assisted breeding programs. Moreover, we observed that photoperiod *Ppd-1* alleles are associated with diverse traits beyond flowering behavior and phenology. Wheat cultivars harboring the insensitive *Ppd-D1a* allele (PpdD1-Hap3) exhibit not only earlier heading, but also deeper roots compared to those having sensitive alleles. Finally, a significant number of modern wheat cultivars were found to carry an approximately 33 Mb *A. ventricosa* segment on the 2A chromosome, linked with the specific PpdA1-Hap1 haplotype which underwent positive selection during 5 decades of wheat breeding in Europe.

### Supplementary Information

Below is the link to the electronic supplementary material.

Supplementary file1 (XLSX 242 KB)

Supplementary file2 (PDF 4467 KB)

Supplementary file3 (FASTA_alignment_Ppd-A1.fas). Multiple sequence alignment of the Ppd-A1 gene generated by ONT(FAS 806 KB)

Supplementary File 4 (FASTA_alignment_Ppd-B1.fas). Multiple sequence alignment of the Ppd-B1 gene generated by ONT (FAS 1111 KB)

Supplementary File 5 (FASTA_alignment_Ppd-D1.fas). Multiple sequence alignment of the Ppd-D1 sequences for 12 reference-quality pseudomolecule assemblies (FAS 145 KB)

## Data Availability

The authors declare that all data are contained within the paper and supplementary information. Ppd-1 gene sequences generated in this study are also available from the NCBI GenBank under accession numbers PP721004–PP721185.
