# Supplementary material for: Novel PHOTOPERIOD-1 gene variants associate with yield-related and root-angle traits in European bread wheat
Source: Theor Appl Genet. 2024 May 10;137(6):125. doi: 10.1007/s00122-024-04634-9 (PMC11087350; doi:10.1007/s00122-024-04634-9)
Supplement: Supplementary file 2 — Supplementary file2 (PDF 4467 KB) [file 122_2024_4634_MOESM2_ESM.pdf]

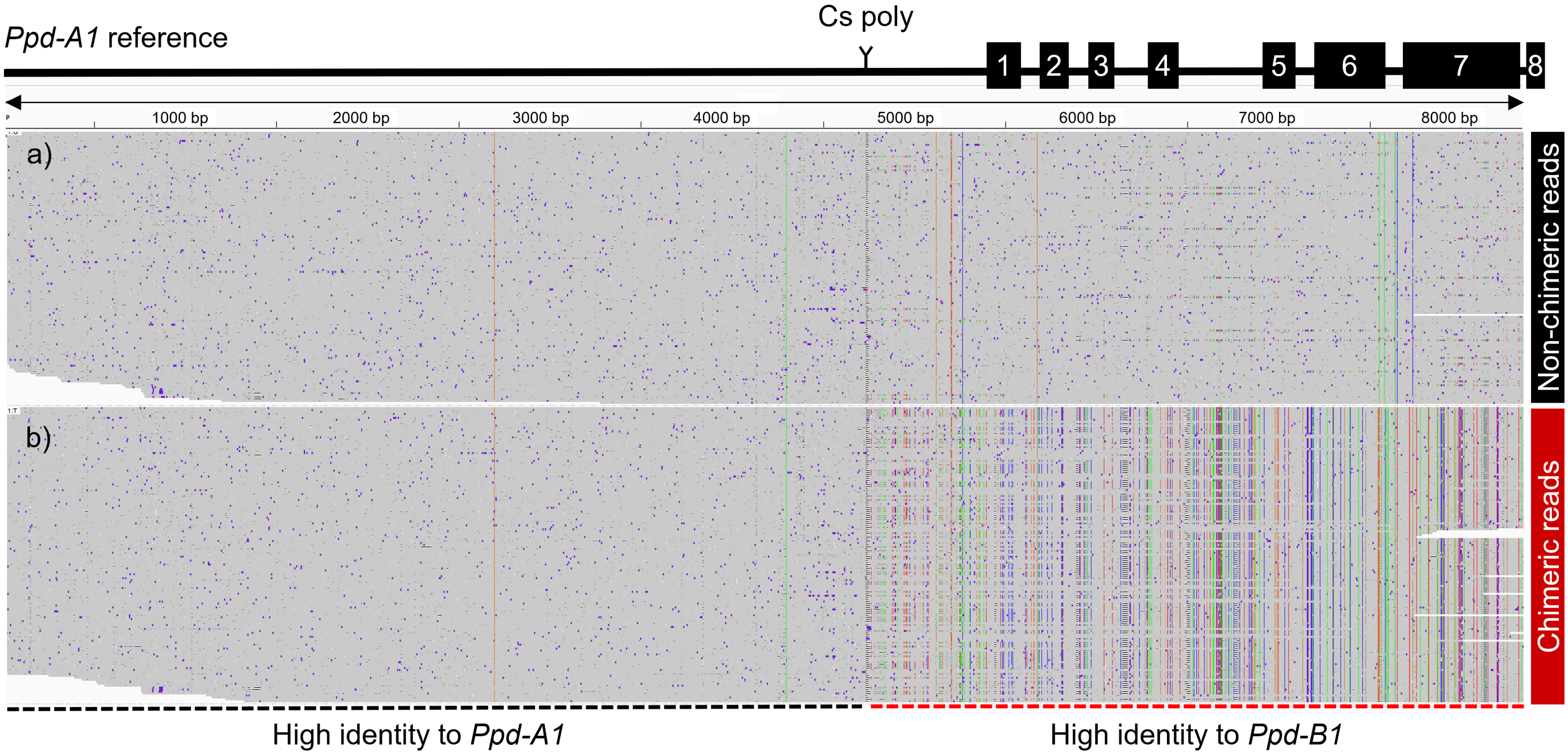

Supplementary Fig. 1 Visualization of ONT read mapping to *Ppd-A1* reference for one example cultivar in the Integrative Genomics Viewer (IGV) software. ONT reads were classified into two groups after sorting the alignments based on mismatched nucleotides. a) non-chimeric reads, where the full-length reads exhibit high identity to the *Ppd-A1* reference. b) chimeric reads, composed of two distinct segments. The first segment displays a high degree of similarity to the *Ppd-A1* sequence (black dashed line), while the second segment exhibits a high similarity to the *Ppd-B1* sequence (red dashed line). In the read alignment tracks, bases that match the reference are shown in gray, while bases with differences are represented by color codes (A, T, C, and G). Deletions are indicated by a black dash (–)

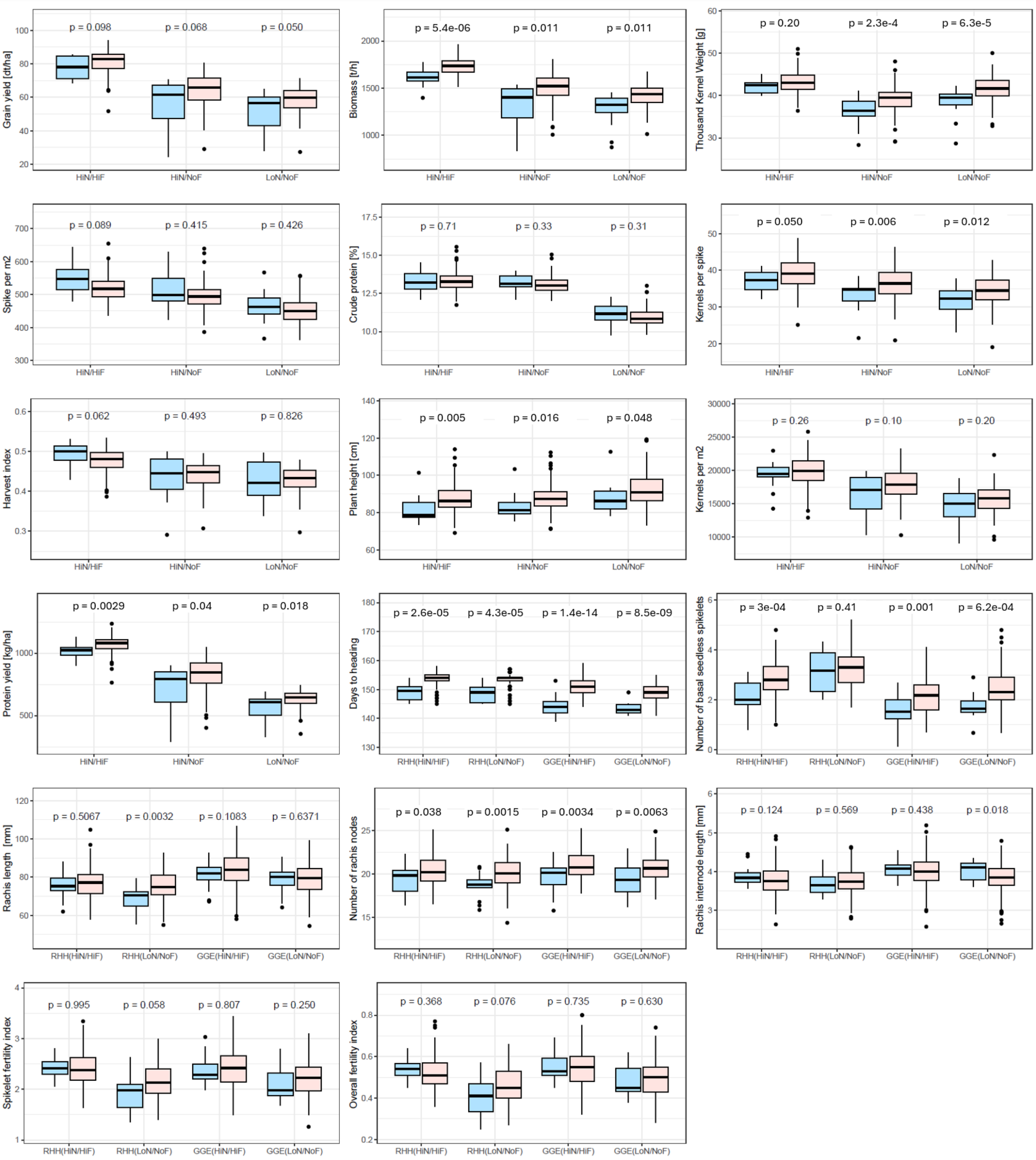

Supplementary Fig. 2 Pairwise comparisons between the insensitive PpdD1-Hap3 haplotype of the *Ppd-D1* gene (represented by the blue box) and other sensitive haplotypes, PpdD1-Hap1 and PpdD1-Hap2 (represented by the pink box), and their associations with 17 agronomic traits across various German environments, including the corresponding p-values. The experiment includes three agrochemical treatments: high nitrogen with fungicide (HiN/HiF), high nitrogen without fungicide (HiN/NoF), and low nitrogen without fungicide (LoN/NoF). The RHH and GGE represent Rauischholzhausen and Gross-Gerau locations, respectively.

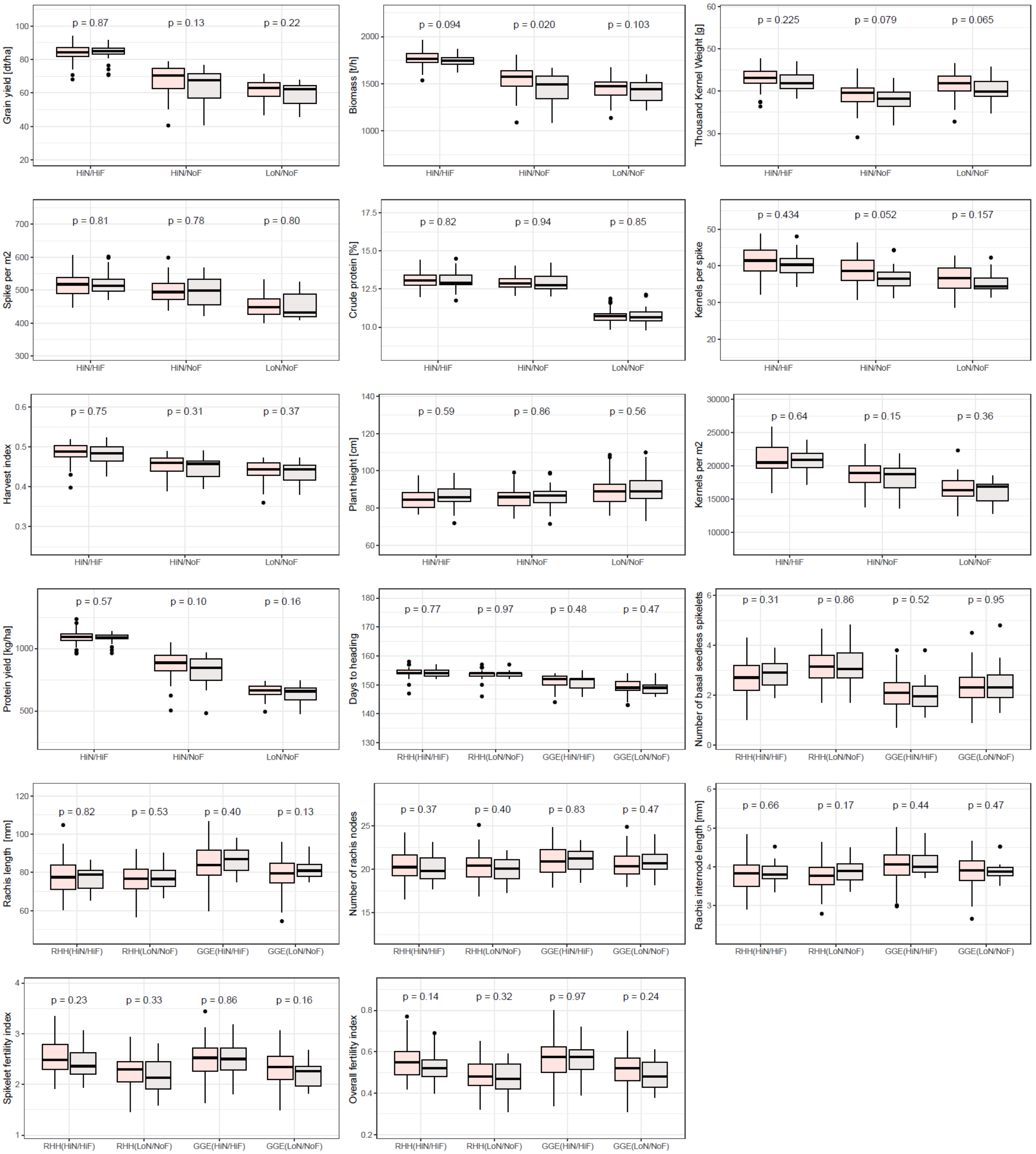

Supplementary Fig. 3 Pairwise comparisons between the two groups of *Ppd-D1*, group one comprises cultivars carrying the combination PpdA1-Hap1 plus PpdD1-Hap1 (depicted by the pink box), while group two represents cultivars carrying the combination PpdA1-Hap1 plus PpdD1-Hap2 (depicted by the gray box). These comparisons were made in association with 17 agronomic traits across various German environments, with the corresponding p-values. The experiment includes three agrochemical treatments: high nitrogen with fungicide (HiN/HiF), high nitrogen without fungicide (HiN/NoF), and low nitrogen without fungicide (LoN/NoF). The RHH and GGE represent Rauischholzhausen and Gross-Gerau locations, respectively. Both groups exhibited a single copy of *Ppd-B1*

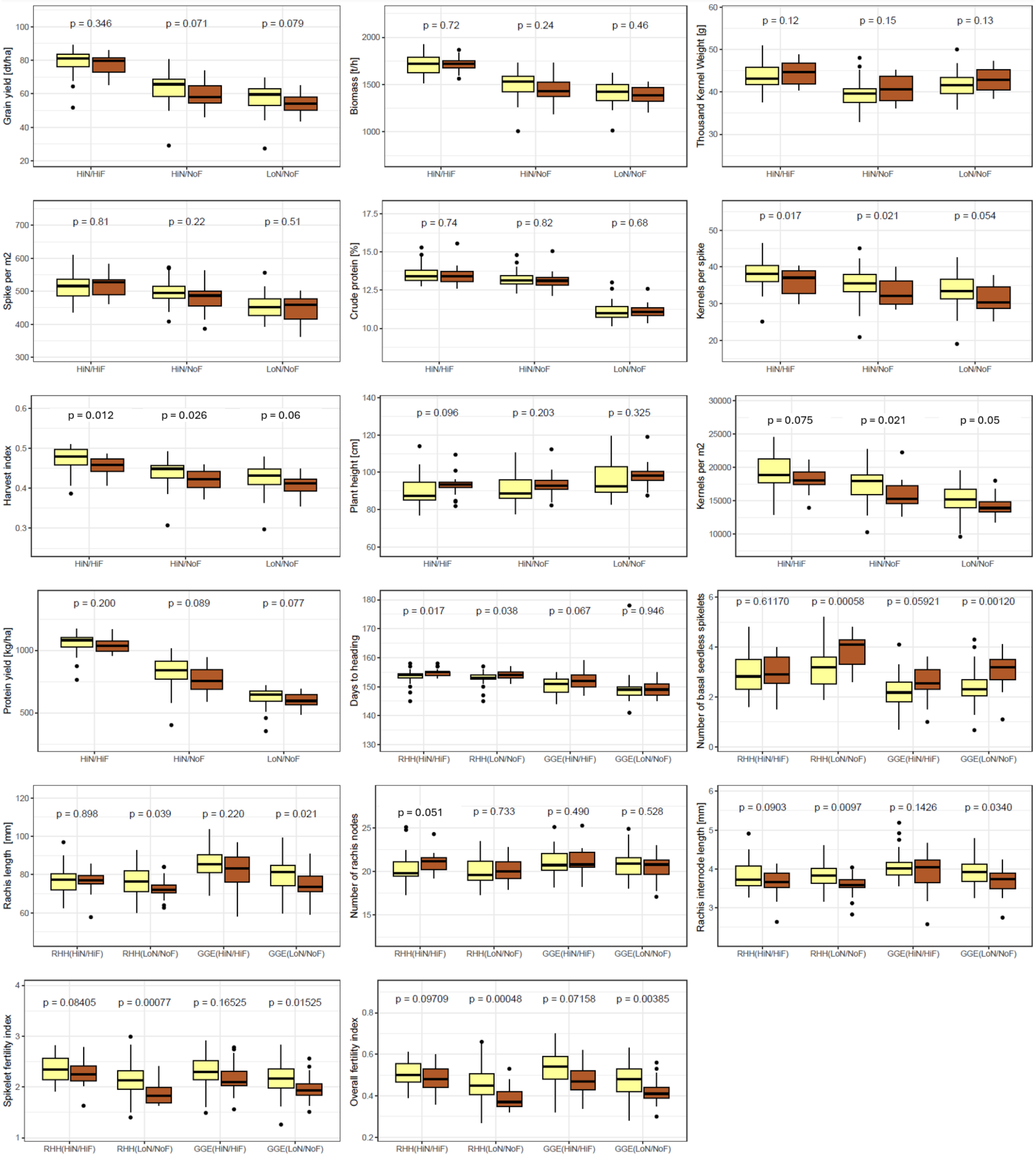

Supplementary Fig. 4 Pairwise comparisons between the two groups of the *Ppd-B1* gene, Group one comprises cultivars carrying the combination of PpdA1-Hap4 plus PpdB1-Hap1(represented by the yellow box), while group two consists of cultivars carrying the combination PpdA1-Hap4 plus PpdB1-Hap2 (represented by the orange-brown box). These comparisons were made in association with 17 agronomic traits across various German environments, with the corresponding p-values. The experiment includes three agrochemical treatments: high nitrogen with fungicide (HiN/HiF), high nitrogen without fungicide (HiN/NoF), and low nitrogen without fungicide (LoN/NoF). The RHH and GGE represent Rauischholzhausen and Gross-Gerau locations, respectively. Cultivars carrying the insensitive *Ppd-D1a* allele (PpdD1-Hap3) were excluded from this analysis.

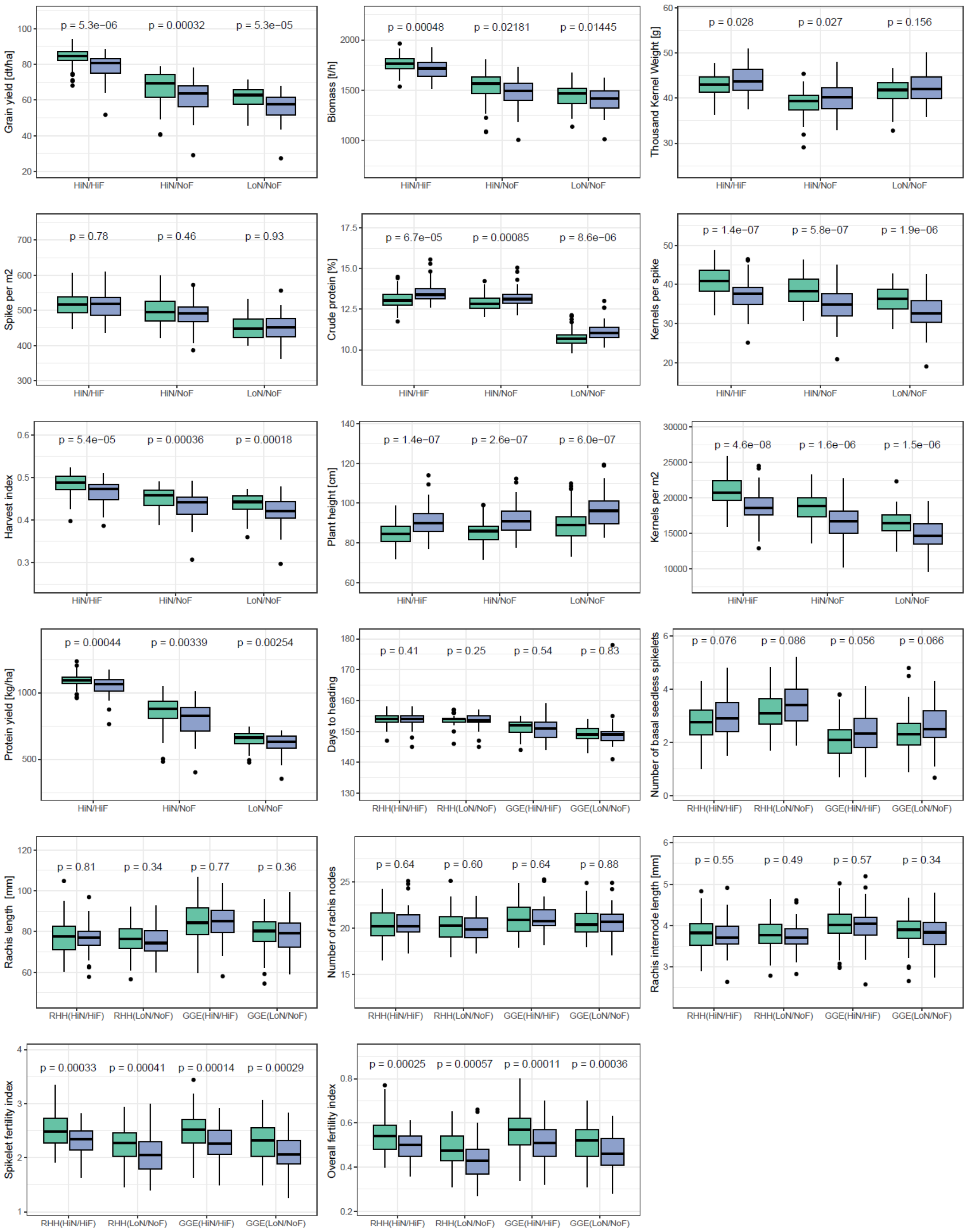

Supplementary Fig. 5 Pairwise comparisons between the two main sensitive haplotypes of the *Ppd-A1* gene, PpdA1-Hap1(represented by green box) and PpdA1-Hap4 (represented by blue box), and their associations with 17 agronomic traits across various German environments, with the corresponding p-values are provided. The experiment includes three agrochemical treatments: high nitrogen with fungicide (HiN/HiF), high nitrogen without fungicide (HiN/NoF), and low nitrogen without fungicide (LoN/NoF). The RHH and GGE represent Rauischholzhausen and Gross-Gerau locations, respectively. The cultivars carrying the insensitive *Ppd-D1a* allele (PpdD1-Hap3) or multiple copy number of *Ppd-B1* (PpdB1-GT4) were excluded from this analysis.

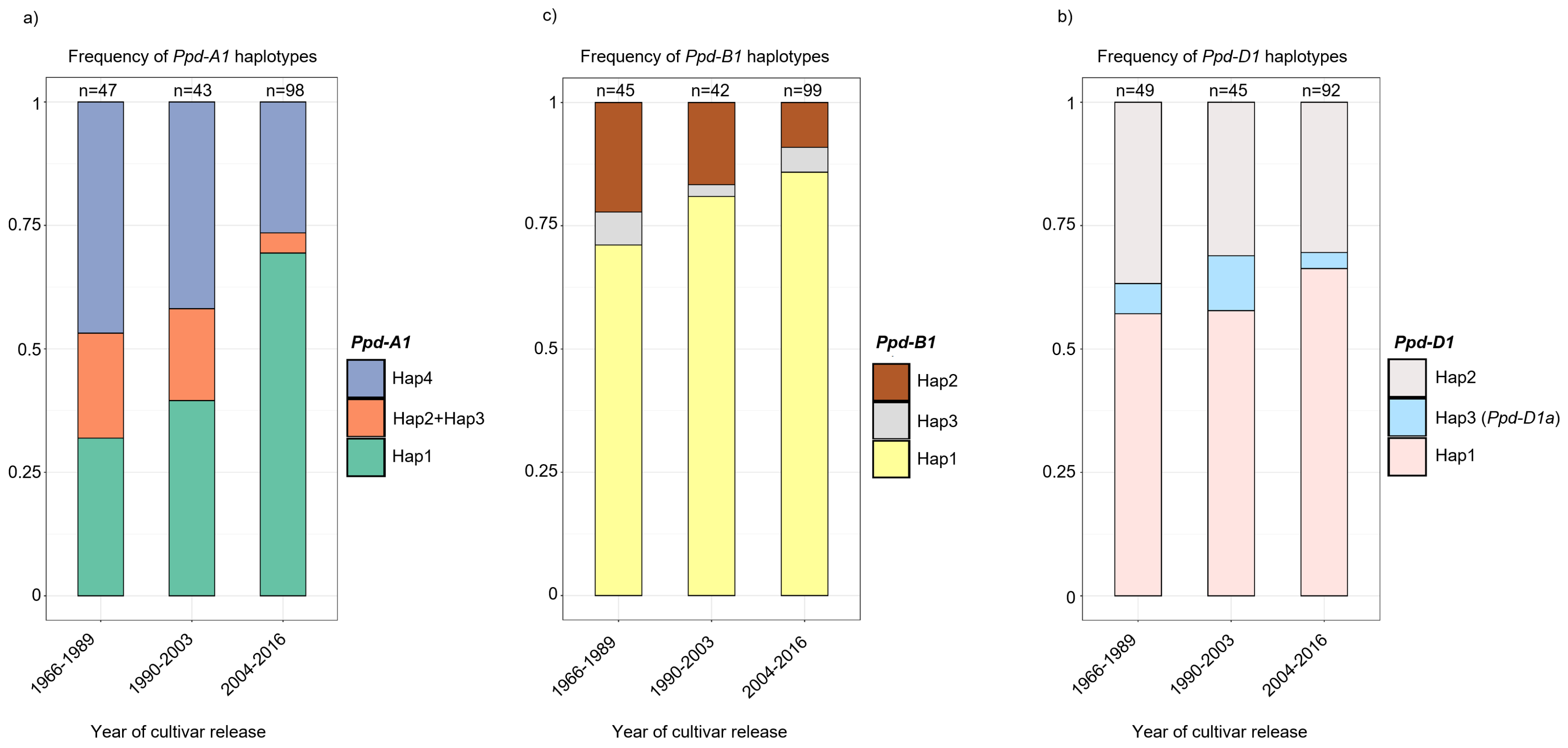

Supplementary Fig. 6 Comparison of the frequency of *Ppd-1* haplotypes within a diverse panel of 192 wheat cultivars released over a period of 50 years of breeding. a) Frequency distribution of *Ppd-A1* haplotypes. b) Frequency distribution of *Ppd-B1* haplotypes. c) Frequency distribution of *Ppd-D1* haplotypes. The number above each bar indicates the count of cultivars for each time group.
